# 

**Published:** 1879-10

**Authors:** 


WITH the September number, we enclosed to each subscriber a bill for the annual subscription, in accordance with our terms "IN ADVANCE." The response to this appeal has been generally prompt, for which we beg to return our grateful acknowledgments. We do not wish to be understood, also, as insinuating that the few on our list who have failed to respond to our call intend any indifference to our efforts to furnish a first-class journal, or any desire to discourage us in our work. We prefer to think that the habit of procrastination, peculiar to many members of the profession, may explain their delay. This, however, if persistently followed, may embarras us, at no distant day, in responding to the polite note we receive monthly from our printer, to call at his office and settle. While assuring our subscribers of our forbearance towards their omissions, and our aim to deal generously, and honestly and justly with them, we trust they will second our efforts, by remitting, as promptly as circumstances will permit, the amount due.

The warm reception extended by the profession to the present volume, and the favorable criticisms of the medical and secular press, together with the rapid increase of our circulation, now double that of the previous volume, are sources of encouragement, which it will be our earnest effort to merit, by an endeavor to improve the JOURNAL, with each issue. Already we have been compelled to augment its size, by an increase of the number of pages, and by the addition of a new section, which our readers will observe in the Clinical Reports, to which we direct their special attention, and also invite their co-operation. The report of interesting cases, occurring either in general or hospital practice, will be of great value to the general practitioner, who seeks practical hints, to be utilized in his daily duties, rather than elaborate papers, which require more uninterrupted leisure than is often at his command.

It is confidently expected that in thus endeavoring to merit the patronage of the medical profession in every section of the country, we will contribute somewhat to that progressive spirit which keeps medicine in the vanguard of scientific research.

### BOOKS AND PAMPHLETS RECEIVED.

**A Treatise on Hygiene and Public Health.** Edited by ALBERT H. BUCK, M. D., American editor of Ziemssen's Cyclopædia of the Practice of Medicine. New York: William Wood & Co.

**The National Dispensatory.** STILLE & MAISCH. Second edition. Philadelphia: Henry C. Lea.

**Analysis of Urine.** By HOFFMAN & ULTZMAN. Translated by T. B. BRUNE, A. M., M. D. New York: D. Appleton & Co.

**Summer and its Diseases.** By JAMES C. WILSON, M. D., Physician to the Philadelphia Hospital, &c. Philadelphia: Lindsay & Blakiston.

**Student's Pocket Medical Lexicon.** By ELIAS LONGLEY. Philadelphia: Lindsay & Blakiston.

**Eye Sight and How to Care for it.** By GEO. C. HARLAN, M. D. Philadelphia: Lindsay & Blakistou.

**Report on Yellow Fever to the St. Louis Medical Society.** By W. HUTSON FORD, M. A., M. D., Professor of Physiology in the New Orleans School of Medicine. St. Louis: Geo. O'Rumbold & Co.

**A Case of Alleged Malpractice, with a Review of the Medical Testimony.** By HOMER O. HITCHCOCK, A. M., M. D., of Kalamazoo, Mich.

**The Future Influence of the John Hopkins' Hospital on the Medical Profession of Baltimore.** By JOHN VAN BIBBER, M. D.

**North Carolina Board of Health Method for Performing Post-Mortem Examinations.**

